# The link between quality and accreditation of residency programs: the surveyors’ perceptions

**DOI:** 10.1080/10872981.2016.1270093

**Published:** 2017-01-13

**Authors:** Renato Antunes dos Santos, Linda Snell, Maria do Patrocinio Tenorio Nunes

**Affiliations:** ^a^ Department of Education and Health, Faculty of Medicine, University of São Paulo (USP), São Paulo, Brazil; ^b^ Mental Health Unit, University Hospital of Brasília, University of Brasília (UnB), Brasília, Brazil; ^c^ McGill’s Centre for Medical Education, Montreal, QC, Canada; ^d^ Royal College of Physicians and Surgeons of Canada, Ottawa, ON, Canada; ^e^ Discipline of General Practice and Propaedeutic, Department of Internal Medicine, University of São Paulo (USP), São Paulo, Brazil

**Keywords:** Accreditation, residency, medical education, surveyors, program director

## Abstract

Accreditation of medical residency programs has become globally important. Currently it is moving from the goal of attaining minimal standards to a model of continuous improvement. In some countries, the accreditation system engages peers (physicians) to survey residency programs. The surveyors are sometimes volunteers, usually engaged in multiple clinical and education activities. Few studies have investigated the benefits of residency program evaluation and accreditation from the perspective of the surveyors. As peers they both conduct and receive accreditation surveys, which puts them in a privileged position in that it provides the surveyor with an opportunity to share experiences and knowledge and apply what is learned in their own context. The objective of this study is to obtain the perceptions of these surveyors about the impact of an accreditation system on residency programs. Surveyors participated in semi-structured interviews. A thematic analysis was performed on the interview data, and resulting topics were grouped into five themes: Burden (of documentation and of time needed); Efficiency and efficacy of the accreditation process; Training and experience of surveyors; Being a peer; Professional skills and recognition of surveyors. These categories were organized into two major themes: ‘Structure and Process’ and ‘Human Resources’. The study participants proposed ways to improve efficiency including diminish the burden of documentation to the physicians involved in the process and to increase efforts on training programs and payment for surveyors and program directors. Based on the results we propose a conceptual framework to improve accreditation systems.

**Abbreviations:** PD: Program director

## Introduction

Accreditation of medical residency programs has become increasingly important in many countries [1,2]. The goal of accreditation is to attain a minimum standard [3] in order to improve the quality of a program [4,5]. Although there have been calls in the literature for research into accreditation [6] there is scant evidence to support the value and merits of this process [6–9]. As the complexity of both residency education and health care delivery increases [10], the gap between the education and health system data gathered during the accreditation process and daily reality of the residency programs widens. The result is a perception that the data gathered during accreditation may not reflect the quality of a residency program [8,11]. In order to link the accreditation data and the program quality perceived on the ground, a program evaluation process must look at least in part at the final ‘product’, or ‘outcome’.

A number of program evaluation models may potentially inform residency accreditation to define this link. The models developed by Donabedian: ‘Structure, Process, Outcome’ [12,13] and Stufflebeam: ‘Context, Input, Process and Product’ [14] continue to be paradigmatic for evaluation procedures in education and health.

Each model has a result or product that must be evaluated. The ‘product’ in a residency program, ‘a competent physician’, may be very difficult to assess [15].

**Figure 1.**
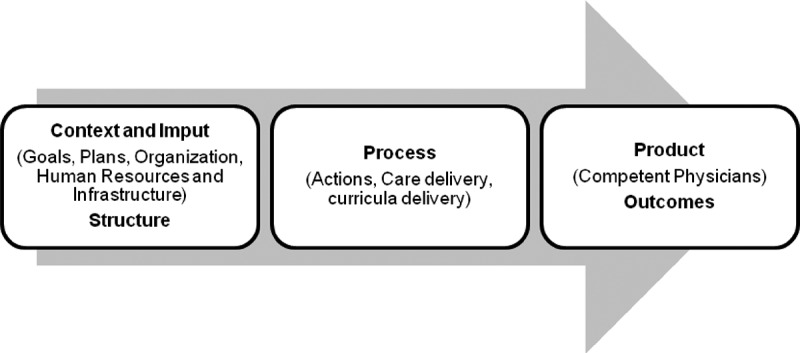
Theoretical framework of health educational program evaluation

In the present study a theoretical framework was developed based on the unification of the evaluation theories of health care and educational programs of Donabedian and Stufflebeam as shown in Figure 1.

One of the main purposes of an accreditation of a residency program is to identify high-quality programs. The factors that contribute to this quality may contribute to a model to assist other programs improve. Accreditation systems must pay attention to the key components of an effective program: working environment, transitions, education to service balance, mentorship, academic curriculum, assessment procedures and the qualities of the program director (PDs) [12].

Despite efforts to define and capture quality in residency programs, the link between the accreditation and quality of physician or patient outcomes is still uncertain [13,14]. Accreditation has been described as a ‘biopsy’ that may have little connection with the routine and daily life of the medical residency [10]. Concerned about this mismatch, the Royal College of Physicians and Surgeons of Canada commissioned a report about stakeholders’ perception of the accreditation process [11]. The report describes positive comments about the rigor and transparency of the accreditation process, and the importance of peers external to the institution who may see ‘what internal eyes may not notice’ [11]. However, this report and others note that the complex accreditation process is perceived as a burden that takes the PDs’ focus away from running their program and training the residents [10,11,15].

In Canada, accreditation surveyors are peers: physicians in practice with experience as teachers, PDs, or with other roles in residency training [16].These surveyors gather data, and as peers they are also in a position of reflecting on these data and applying the information to their own programs. Their perceptions may be able to shed light on the connection or disconnect, between quality as perceived by the stakeholders in the program [11] and quality as captured by other accreditation data, processes and systems. The perspectives of the accreditation surveyors might thus provide information about the relationship between the accreditation process and perceived program quality.

This study aims to clarify the impact of residency accreditation system on the quality of programs from the perspective of the surveyors. The research question is: What are peer-surveyors’ perspectives about the process of residency accreditation in Canada?

## Methodology

This is a qualitative descriptive study [17–19] using semi-structured individual interviews. The main objective of this study was to understand the surveyors’ perceptions of the medical residency programs accreditation process in Canada. Participants had to have at least one experience as a peer surveyor in a residency accreditation process and remain involved in residency training of a surveyed program. A snowball process starting from one single interviewee in one institution was used to recruit the 11 participants. The setting for the interview was the participants’ workplace, with only the presence of interviewer and interviewee.

A semi-structured interview protocol was developed, based on the literature review data, document analysis of accreditation bodies and expert opinions. A pilot test was conducted: the questions were reviewed by faculty members who were experienced in the accreditation process and were subsequently refined. A final semi-structured questionnaire (to guide the interviews) was developed with open-ended questions, giving time and space to the interviewee to explain and ‘tell stories’ around the studied subject. The first author, a physician familiar with qualitative methodology and with no previous relationship with the participants, conducted all the interviews. The interviews were audio-recorded, with field notes made during and just after interview. Each interview lasted about one hour and was transcribed after participant de-identification, with each interviewee represented by a letter and a number (e.g. H.01). Recording the interviews was done to increase quality capture of data and ensure the descriptive validity [20].

The text analysis is based on the method for thematic analysis proposed by Braun and Clark [21,22]. The first author started the content examination by reading and listening to the data to classify and code it through inductive thematic analysis. There was no pre-existing coding framework. A second independent researcher analyzed the data to achieve convergence of opinions. Very few discrepancies were found during this process and when they occurred, they were solved through discussion and consensus. The coding process was repeated iteratively with reading of the transcription and listening to the recorded interviews until no new codes emerged. Saturation of the codes was reached at the seventh interview transcription, but the analysis continued until the eleventh and final interview. The investigators examined data systematically for convergence and divergence in order to develop categories. Categories were added, changed, or removed iteratively until data saturation was reached. Quotes extracted from the interviews were selected for each category demonstrating the richness of data and are in the results section. The relationship between categories was developed into a conceptual framework. A member check was not done.

**Table 1.** Characteristics of participants. n (%).

**Current roles in residency education**	6 (55%) Program director
	6 (55%) Examiner on certifying exams
	5 (45%) Residency program committee member
	3 (27%) Clinical supervisor
**Experience (time) as surveyors**	9 (82%) 1–2 years
	0 (0%) 3–4 years
	2 (18%) > 5 years
**Experience (visits) as surveyors**	7 (72 %) 1–3 visits
	2 (18%) 4–6 visits
	2 (18%) 7–12 visits
**Years in practice**	1 (10%) < 5 years
	0 (0%) 6–14 years
	5 (45%) 15–19 years
	5 (45%) > 20 years

McGill’s University institutional research ethics board approved this project. Following explanation, each interviewee signed a consent form.

## Results

Among the twenty-one named physicians eleven fulfilled the inclusion criteria of having been both surveyed and a surveyor for a residency accreditation process (Table 1). The surveyors were from one institution but had surveyed many other residency programs across Canada. They had years of medical practice experience, current roles in residency education (examiner on certifying exams; residency program director; residency committee member and/or clinical supervisor) beyond experience as a visiting program surveyor (Table 1).

From the thematic analysis six categories emerged: Training and experience; Being a peer; Professional skills and recognition as a surveyor; Burden (documentation and time); and Efficiency and efficacy of the accreditation process; (Table 2); and new ideas to improve efficiency (Table 3).

**Table 2.** Thematic analysis.

Primary level Coding	Category or concepts	Theme
Little organization of surveyor training	1. Training and experience	
Learning from experience		
Mentorship by surveyors		
Learning opportunity for the surveyor		
Surveyor experience teaches how to be a better PD		
Advantages of having been surveyed as well		
Importance of the peers in all steps	2. Being a peer	HUMAN RESOURCE
High level of self-requirement		
PDs’ impact on the programs		
Peer presence impacts quality		
Peers role in residents evaluation		
Surveyor need experience as a PD		
Surveyors lacks professional ‘survey’ skills/expertise	3. Professional skills and recognition	
Problems with a volunteer model		
Lack of financial or other benefit to surveyor		
To be a surveyor is time consuming		
Stress	4. Burden (documentation and time)	STRUCTURE AND PROCESS
Concern about cost		
Unnecessarily time consuming		
Burden of documentation		
Things could be made ‘lighter’		
Schedule extremely tiring		
System needs to be changed	5. Efficiency and Efficacy of the Accreditation Process	
Not enough qualitative data		
Problem with focus: does not capture data for quality		
Emphasis on process, not outcomes		
Lack of efficiency		
The accreditation visit seems to be a theater		
Discrepancy between information provided and reality		
Not enough capacity do capture quality		
Gather only meaningful data

**Table 3.** New ideas to improve efficiency.

Separate peers’ and professional surveyors’ evaluations
Split evaluation of administrative structure and faculty from the evaluation of individual programs
Utilize physicians and non-physicians, both specialists in Medical Education having mandates inside the accreditation system
Increase engagement of the specialty committees
Separate ongoing review of documentation and periodic on-site reviews.

The first five categories were organized into two major themes:

*‘Human Resources’ and ‘Structure and Process’.* 

The peer position of the participants brought their perceptions both as surveyors and as those being surveyed. For this reason, although our questions were about the surveyors’ perceptions of residency accreditation, the responses and categories often reflected the PDs' perceptions also. Several comments from PDs perceive the accreditation as a test of their performance, such as the following example:

‘If I had a bad accreditation outcome when I was running the residency program, I would have been prepared to resign’ *[I. 05]*

Participants perceived the residency accreditation process as a powerful system with positive impact on the quality of the programs. The pure existence of a set of standards and a system to verify them brings benefits to all. Nevertheless, the process is seen as a burden, with problems in efficiency and in recruiting and developing the needed human resources. All participants thought that system needed changes, as exemplified by the following text:

‘… in everybody’s mind, it’s really powerful when the [accreditation] comes to your place’ [I.05]

### Human resources

There is consensus among participants about the importance of the peer in the core of the process. Someone who had been a resident and is now engaged in training residents is perceived as abler to judge and analyze another residency.

‘Personally I think I like the idea that it’s a colleague that does

It, in the way that they understand my reality somehow’ [I. 09]

However, there is a perception among the participants that experience and further training is needed to develop skills as a qualified surveyor.

‘The training process is minimalist to say the least … I do think that the preparation is inadequate, especially for your first accreditation’ [I.08]

Participants perceived that the quality of a program captured by the accreditation depends on how well a surveyor gathers, integrates, and reports the data. Surveyors have suggested that an ongoing training program addressing theoretical and practical aspects of accreditation would help increase surveyor competency. Interviewees thought that groups of well-trained, experienced professionals who are dedicated to the task of become a surveyor would improve the system.

Participants recognized that efforts are being made to build high standards and acquire required data. Nevertheless, from the human resources point of view there is a perception that little is being done to build capacity or to retain experienced people. They perceived a lack of activities such as mentoring by experienced surveyors, active learning, and learning while doing the survey.

‘I think you have to teach the surveyor before they survey. You keep a bank of surveyors, they should take a course every two or three years, but at the same time they should be paired with an experienced surveyor the first time’[I.10]

However, at the same time they recognize that physicians usually do not have time to develop surveyors’ skills. They also recognize that having people devote enough time to the task of surveying may not be feasible in a volunteer system, which ends up decreasing the likelihood of having experienced surveyors. Usually individuals volunteer to do a survey once, twice, or rarely more than six times in a lifetime.

The residents’ interview during an onsite survey is an example of the importance of training and experience. The interview with a group of residents was described as the most ‘precious’ yet challenging moment. The surveyor must be able to capture what residents have said, what the program has done, and weigh the merits of each.

‘I’ve realized that the perception of the residents is the key. So [as PD] I’m very sensitive to that now, when my residents are unhappy I solve this right away. That’s what kills you [in an accreditation]’ *[I.09]*

### Structure and process

The majority of the participants believe that some of the required documents and data could be removed from the surveyor analyses to gain efficiency without losing efficacy. Participants perceived that the accreditation system has been increasing its requirements for data, resulting in increased surveyor time and energy. Through participants’ comments it is possible to deduce that this heavy ‘growing machine’ can lose focus and require data that does not contribute to accreditation decisions. Time needed by the PDs to complete the forms and pre-survey questionnaires, the amount of documentation to be read before an onsite visit by a surveyor, and the onerous agenda during onsite visits were cited as examples of unnecessarily time-consuming tasks. The aforementioned time could be spent in a better way.

‘That experience I found very time consuming and the idea of working until 2 am doesn’t work for me…I was thinking that it was such a waste of my time. It’s not a good process…’*[I09]*

According the participants, the accreditation process might end up as a burden from the perspective of the surveyors and of those surveyed. As an example, before an onsite visit a surveyor might receive more than 1000 pages of documentation to analyze in about a week. The comments of the participants suggest that the weight given to this data makes the process stressful.

‘It’s a lot of effort and stress and documentation’ [I.03]

### New ideas

Participants proposed new ideas to improve the system (Table 3). Many of these ideas addressed similar issues or solutions, so they are collected in this section.

The ideas aim to add expertise, making the accreditation ‘lighter’ (by reducing the documentation sent in for review), and allow more focus on outcomes. The ‘best of each world’ idea was well accepted: to blend components of the Canadian system and the Accreditation Council for Medical Graduate Education (ACGME) model by combining professional surveyors and peer surveyors as complementary.

‘I don’t think the whole process has to be done by physicians.’[I.03]

Professional surveyors could maintain the focus on quantitative data and the general standards.

‘I think it’s a great idea to have a professional accreditor, and it wouldn’t even have to be done at the same time. They are probably better at it than we are because they are experienced’[I.08]

Peers would be responsible for the evaluation of the clinical and educational aspects through qualitative data gathered in independent cycles of on-site visits.

Another suggestion was to change the way the data is gathered from a cyclical process to one of ongoing review.

‘like there is someone in the university whose job it is to continuously to apply standards and so on’[I.05]

General standards that are common to all programs (e.g. information about faculty and administrative information) could be obtained and evaluated by a professional surveyor (not a peer). These data would be focused in quantitative and ongoing feeding data, online, with independent cycles of visits. This database would be source of information for specialty committee and surveyors if necessary.

Most participants agreed on the importance of the role of the specialty committee (a national specialty-specific committee which is responsible for creating standards e.g., Radiology, Neurology). The roles of committee members could change. For example, they could analyze the pre-survey data and ‘filter’ what really matters and what requires special attention, turning the actual survey process more efficient.

## Discussion

Accreditation, quality, and continuous improvement have become an intrinsic part of the discourse and activities of health services and health education. Internationally, dating from 1970s, health care accreditation programs and accrediting organizations emerged and developed. A systematic review by Greenfield and Braithwaite concluded that there are two consistent categories of findings as result of accreditation of health services: promoting change and professional development; however there were insufficient studies on issues faced by surveyors [8,23].

Recent research has been made linking the accreditation process and development of medical education, health system, residents’ competencies, patients’ safety and surveyors’ development [23,24]. The vital importance of surveyors’ perceptions inside the residency accreditation was recognized in the present study. Participants perceived that accreditation is related to the quality of residency programs.

Our results suggest a number of ways of improving accreditation systems. Some of these have been adopted in some countries, but not all within one accreditation system. Examples include using professional surveyors and the ongoing process to gathering data (ACGME system) [25]; using peer-surveyors (the RCPSC accreditation system) [4]; and lighter processes (other countries) [26]. Our data regarding surveyors and the survey process are aligned with the literature and principal strategies currently used to improve accreditation systems. The results showed the different perceptions of a national accreditation system, in particular the advantages of having peer surveyors and the challenges and burdens of the current system.

The RCPSC and ACGME [25,27] are currently revising their accreditation systems. The goals of the process are to evaluate, improve, and publicly recognize graduate medical education programs and their sponsoring institutions. This will ensure substantial compliance with the standards of educational quality established by the ACGME, in order to protect the interests of residents, their teachers, and clinicians; improve the quality of teaching, learning, research, and professional practice (patient safety).

**Figure 2.**
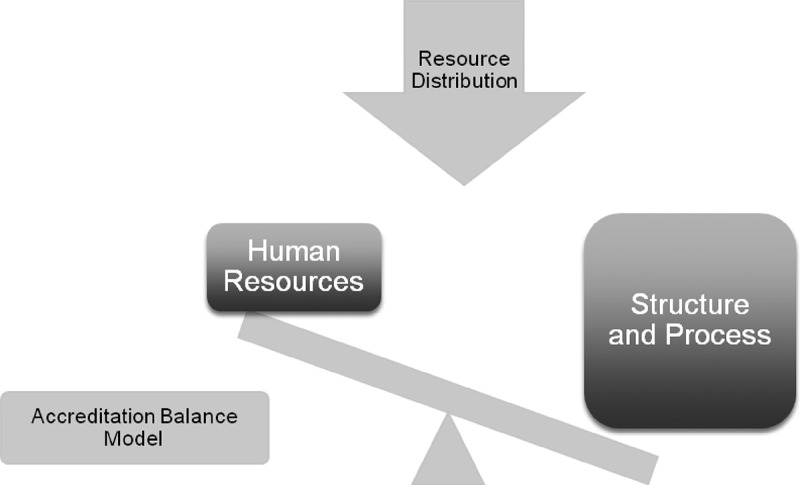
The accreditation balance model

A model (Figure 2) emerged from the data helping to organize the concepts arising from this study. The model explains the importance of a balanced investment of resources as a goal of the system – the ‘Accreditation Balance Model’. On one-hand human resources (surveyor training, expertise, skills in surveying, recognition) is perceived as receiving few resources and little support. On the other hand, the weight put on structure and process (amount and breadth of focus of the documentation, the time and effort needed) has been perceived as a heavy burden to everyone (Figure 2). The goal must be to achieve a better balance between the two.

**Figure 3.**
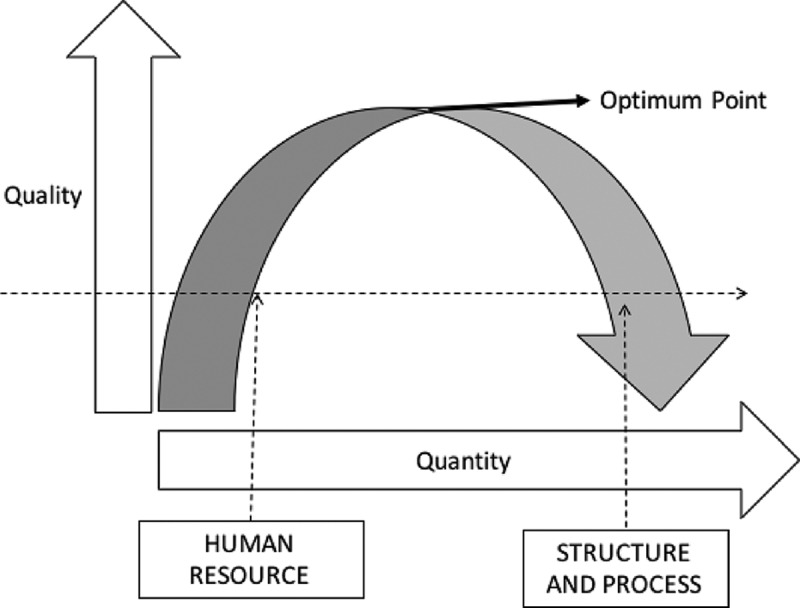
The optimum point

However, by increasing the amount of data gathered, the system initially seems to improve performance, but after an ‘optimum point’, the performance of the system starts to fall. This may happen because there is more to do, and not enough time or human resources to do it (Figure 3).

Physicians usually do not dedicate themselves full time to train or to gain professional skills as fulltime surveyors. However, dedicating time to train or to gain surveyor professional skills might mean losing the peer status.

Poor recognition by institutions of the peers and of the volunteer nature of the system may affect surveyors’ recruitment and consequent experience. The training process for the peers and the recognition of their work also needs balance to achieve the optimum point as shown in a framework developed by the authors (Figure 3).

The accreditation process is an essential part of a quality education program. This study was designed to contribute to improving this process, by adding the perspective of surveyors. The current imbalance between human resources and structure and process shows where the system can be improved.

### Limitations

The small sample size might be considered a limitation to this study, however there was alignment within the participants’ interviews and saturation of the data was found after the seventh interview with no new codes after that. Although data was collected from surveyors in one university in a single country with one accreditation model, these surveyors had performed surveys at many other institutions within Canada. We feel that our concepts of ‘the accreditation balance model’ and ‘the optimum point’ are general enough that they may be applicable in many different accreditation systems.

## Conclusion

Accreditation of residency programs is evolving, however the field needs more study in order to contribute to evidence-based quality improvement. Balancing the investment in the accreditation system between structure and process, and human resources, valuing all individuals involved, and utilizing peer surveyors to focus on relevant data that they can optimally obtain will most likely improve an accreditation process. Exploring peer-surveyors’ perceptions brings valuable information from the field to policy makers and accreditation bodies that may inform future design of an accreditation system.
